# OA04 Paediatric APS - children are not just small adults

**DOI:** 10.1093/rap/rkac066.004

**Published:** 2022-09-28

**Authors:** Rosie Close, Alice Leahy, Kerstin Nott

**Affiliations:** Southampton Children's Hospital, Southampton, United Kingdom; Southampton Children's Hospital, Southampton, United Kingdom; Southampton Children's Hospital, Southampton, United Kingdom

## Abstract

**Introduction/Background:**

Antiphospholipid syndrome (APS) is a rare autoimmune multisystem disease characterised by thrombosis and pregnancy morbidity in the presence of persistently elevated titres of: lupus anticoagulant, anticardiolipin and/or anti-glycoprotein 1. It may be primary (occurring alone) or secondary (in combination with another disease, most commonly systemic lupus erythematosus (SLE)). Recent publications highlighted clinical criteria limitations for children and raised awareness of the burden and prevalence of non-criteria manifestations in this population. This case report adds further weight to the need to raise multi-specialty awareness of non-criteria manifestations to aid recognition and treatment of this rare condition with potentially severe sequelae.

**Description/Method:**

13-year-old female with SLE diagnosed aged 8 in India with bilateral optic neuritis occurring two months later. ANA positive at diagnosis with low complement and thrombocytopenia. Treated with prednisolone and hydroxychloroquine. Patient moved to the UK aged 9; initial abnormal bloods: mildly positive ANA (ENA negative), thrombocytopenia, strong lupus anticoagulant. As serology not strongly suggestive and optic neuritis rare in lupus diagnosis questioned. Ophthalmology review confirmed bilateral optic atrophy without evidence of previous vasculitis. There was debate whether the post-retinal demyelination was due to antiphospholipid syndrome or a primary demyelinating condition. Hydroxychloroquine stopped and azathioprine started. Following normal neurology investigations (brain, spine MRI/MRV/MRA) concluded if patient developed new APS-related symptoms or worsening visual evoked potentials anticoagulation would be discussed. Patient remained stable over four years with chronic thrombocytopenia and ESR persistently elevated. Azathioprine changed to Mycophenolate mofetil (MMF) due to side effects.

Routine medication monitoring bloods in 2022 showed ESR 97, CRP 78, Platelets 61. Review identified vasculitic rash on soles of both feet with palpable nodules and normal pulses. Further investigation confirmed antiphospholipid antibody triple positivity. Aspirin commenced, hydroxychloroquine restarted, MMF dose increased and rituximab administered. Left foot rash settled but right progressed with toe discolouration and numbness. Skin biopsy considered but not performed due to skin integrity concerns. Foot pulses remained present and normal. Bilateral lower limb doppler reported as normal; increased symptoms resulted in CT angiogram which revealed bilateral non-occlusive popliteal thrombus and left pulmonary embolus. Subsequent echocardiogram was normal. Patient was anticoagulated with low molecular weight heparin followed by warfarin. Vascular surgical team advocated medical management and patient received seven infusions of Iloprost followed by Sildenafil. She achieved near total resolution of skin changes to toes with only minimal loss of skin over tip of right great toe. Patient will now require long-term anticoagulation.

**Discussion/Results:**

APS was considered in initial differential diagnosis but patient did not meet current clinical criteria as no past evidence of thrombosis. Lupus anticoagulant was consistently strongly positive and anticardiolipin repeatedly negative. As anti-B2 glycoprotein 1 antibody is not routinely tested and must be verbally requested, it was only checked once (negative) prior to discovery of triple positivity.

ANA reported as strongly positive at time of SLE diagnosis but reviewing original notes from India titre was 1:100 and therefore not highly convincing. ENA negative and complement and white cell count normal on repeat testing since. Therefore, it is probable that this patient has primary APS as opposed to secondary APS in association with SLE. However, it is possible that this patient may develop more symptoms of SLE over time.

When this patient presented with foot rash there were high numbers of children presenting with varying severity of painful, itchy toes coined ‘covid toes’ due to suspected link to SARS-CoV-2 infection. Patient had exposure history, and COVID antibody serology was difficult to interpret due to recent vaccination. Dermatology found appearance to be consistent with ‘covid toes’ and advised supportive treatment. The triple APS antibody positivity result provided probable aetiology. Providing evidence of thrombus was problematic with false reassurance from apparently normal lower limb arterial doppler when actually popliteal arteries were not checked in view of the presence of normal flow proximally at the groin and distally in the feet.

This case highlights the need to continue to search for thrombus in presence of high titres antiphospholipid antibodies and particularly in the case of triple positivity as although patient presented with colour change to toes, she was entirely asymptomatic from her PE and her left foot improved spontaneously despite a left popliteal thrombus also being present.

**Key learning points/Conclusion:**

Non-criteria manifestation of thrombocytopenia (occurs in 25% paediatric APS patients) was present throughout and patient had past history of haematuria (a recognised renal non-criteria manifestation). A paediatric specific APS criteria including these may have resulted in earlier detection of triple antiphospholipid antibody positivity and thus earlier treatment escalation and possible avoidance of thrombus.

It has been reported that a high proportion of children with positive antiphospholipid antibodies don’t develop a thrombus. However, it is interesting that our patient was entirely asymptomatic from her pulmonary embolus which was an incidental finding on her CT angiogram. This prompts a discussion about how much imaging should be performed in those with high levels of persistent positive antiphospholipid antibodies.

Rituximab resulted in normalisation of platelet count and ESR for the first time since initial presentation. Anticardiolipin antibodies normalised, lupus anticoagulant decreased from strong to moderate and anti-B2 glycoprotein levels decreased but remained positive. Rituximab is a recognised treatment for catastrophic antiphospholipid syndrome (CAPS) but not routinely used in APS. The consistently raised ESR in an apparently clinically well patient is a reminder to continue to search for causes of inflammation. As the CRP was largely in normal range, this demonstrates the unique value of the ESR.

In view of anti-B2 glycoprotein 1 antibody requiring to be verbally requested, discussions are ongoing with the laboratory department regarding the possibility of electronic request and a comment with recommendation to check other two antiphospholipid antibodies following one positive antibody result.

As a result of this case, a plan will be put in place to ensure annual screening of antiphospholipid antibodies in all juvenile SLE patients in our care.

It is hoped that this case report promotes discussion amongst the paediatric rheumatology community regarding further research required for development of paediatric specific APS criteria and management.

